# Sandwich mesh repair for the treatment of lumbar incisional hernia: a case series

**DOI:** 10.1186/s12893-026-03679-5

**Published:** 2026-05-20

**Authors:** Yasser A. Orban, Yasser Baz, Yasmine Hany Hegab

**Affiliations:** 1 Department of General Surgery, Faculty of Medicine, Zagazig University, Zagazig, Egypt; 2 Department of General Surgery, Faculty of Medicine, Helwan University, Cairo, Egypt

**Keywords:** Sandwich, Mesh, Lumbar Incisional Hernia, Repair

## Abstract

**Background:**

A variety of surgical techniques have been described for the open repair of lumbar incisional hernias. Determining the optimal surgical technique for these hernias remains challenging. This study evaluates the sandwich mesh technique, preperitoneal mesh, and onlay mesh for the treatment of lumbar incisional hernia.

**Methods:**

This is a prospective study conducted to repair lumbar incisional hernias. We employed the sandwich approach with preperitoneal and onlay mesh reinforcement. Postoperative outcomes, complications, and length of hospital stay were assessed. The primary outcome was recurrence of hernia, and secondary outcomes included wound seroma, surgical site infection, and wound ischemia.

**Results:**

Ten patients were involved in this study, six males and four females, with a mean age of 50.5 ± 9.32. The main complaints were abdominal protrusion at a site of previous abdominal operation, both in 100% of cases. Pain was present in four patients. All the patients had previous surgery through a lumbar incision; the most frequent surgery was for ureteric stone (7 patients), while the other cause was for nephrectomy (3 patients). 50% of patients presented with comorbidities, including diabetes mellitus, smoking habits, ischemic heart disease, and hypertension. The operative time was 76.4 ± 11.86 min. The mean hospital stay was four days. Complications were infrequent and manageable, with seroma formation being the most common. No major morbidity was observed.

**Conclusion:**

The sandwich mesh repair technique represents a potentially feasible surgical option for lumbar incisional hernia repair, demonstrating satisfactory early outcomes and acceptable complication rates.

**Trial registration:**

NCT07196254.

## Introduction

Lumbar incisional hernias are rare abdominal wall defects, accounting for less than 1.5% of all types of abdominal hernias [1]. They typically arise as a postoperative consequence of various surgeries, including nephrectomy, repair of abdominal aortic aneurysms, excision of tumors in the abdominal wall, and plastic reconstructive treatments that include the mobilization of the latissimus dorsi muscle [2]. Incisional lumbar hernias complicate 7% of retroperitoneal approaches [3].

Lumbar hernias are located in the wide anatomic region that is bounded by the external oblique muscle laterally, the erector spinae muscle medially, the iliac crest inferiorly, and the 12th rib superiorly [4]. Lumbar hernias are classified into two groups according to etiology: primary and incisional. Primary lumbar hernias are classified into two subtypes according to their site: the petit subtype in the inferior lumbar triangle and the Grynfeltt subtype in the superior lumbar triangle [5].

Patients diagnosed with lumbar incisional hernia have a bulge in the suprailiac region, situated at the site of the prior surgical incision, and may be accompanied by pain [6].

Surgical intervention remains challenging due to its low prevalence, which results in insufficient experience in managing this pathology, alongside difficulties in clinically delineating the defect’s boundaries and the presence of muscular atrophy and may be nerve injury due to the previous surgical intervention [7].

A variety of surgical techniques have been described for the open repair of lumbar incisional hernias, including simple closure, the use of rotational musculofascial flap secured to bone for defect coverage, fascial strip repair, and the application of synthetic meshes such as polypropylene. Determining the optimal surgical technique for these hernias remains challenging, as no single procedure has demonstrated significant advantages over others in terms of minimizing postoperative recurrence rates [8].

Large abdominal incisional hernias are challenging to be repaired surgically, and recurrence is a frequent complication. There have been reports of recurrence rates as high as 33% after the first repair and 58% following the second [9].

Lumbar incisional hernias are associated with high recurrence rates owing to inadequate fascial support and suboptimal outcomes following conventional repair. This study evaluates the short-term results of a sandwich mesh hernioplasty technique using dual-layer polypropylene reinforcement.

## Patient and methods

This prospective study was conducted to repair incisional lumbar hernias in ten patients. We employed the sandwich approach with preperitoneal and onlay mesh reinforcement. The study was undertaken in the Department of General Surgery at Zagazig University Hospitals from December 2023 to December 2024, after IRB approval and informed written consent from the participating patients.

Patients older than 18 years old with Incisional lumber hernia were included. While, pregnant females, patients with bleeding tendency, patients with complicated hernia and patients who are not fit for surgery were excluded.

**Fig. 1:**
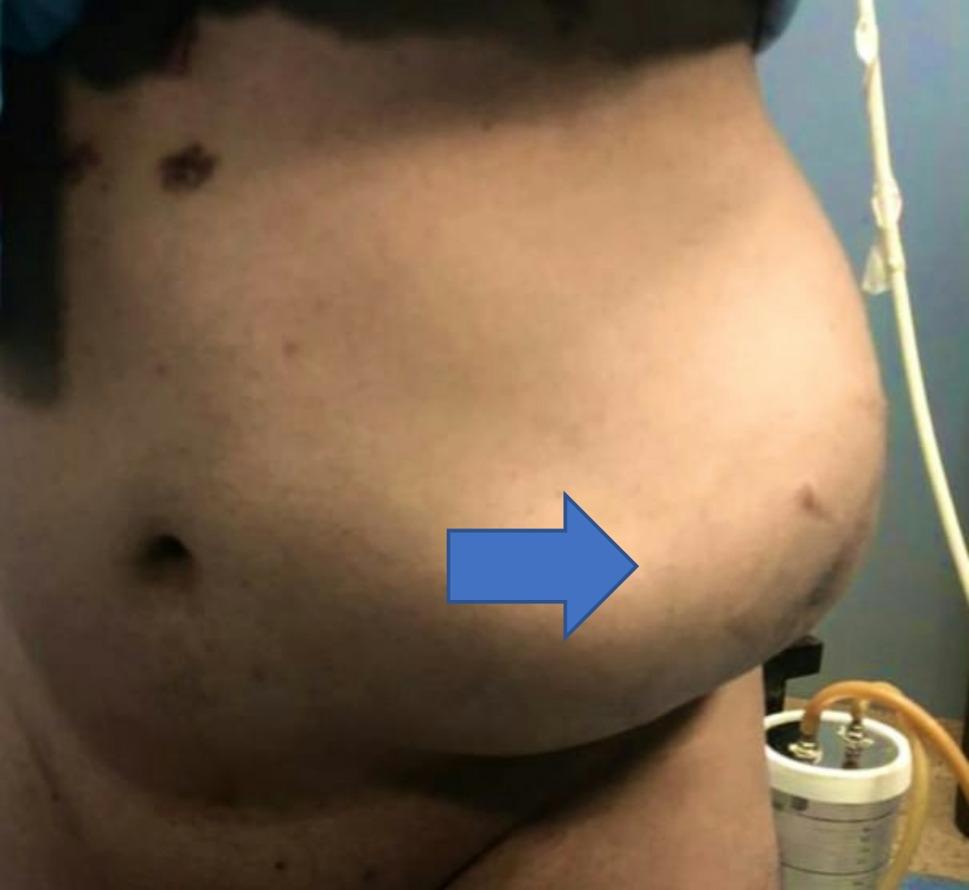
Standing patient with a lumbar incisional hernia, with an arrow pointing to the location of the hernial bulge

The patient underwent clinical evaluation with comprehensive history taking, including surgical and medical histories, and underwent through both general and localized examinations(Fig. 1).

The patients underwent preoperative evaluation and optimization, which included assessing cardiopulmonary functions, cessation of smoking for at least one month prior to surgery for smokers, and regulation of blood glucose levels for diabetics, specifically targeting HbA1c levels less than seven. pelvi-abdominal US and CT scans were utilized to determine the location, size, and number of hernia defects.

## Operative technique

**Fig. 2:**
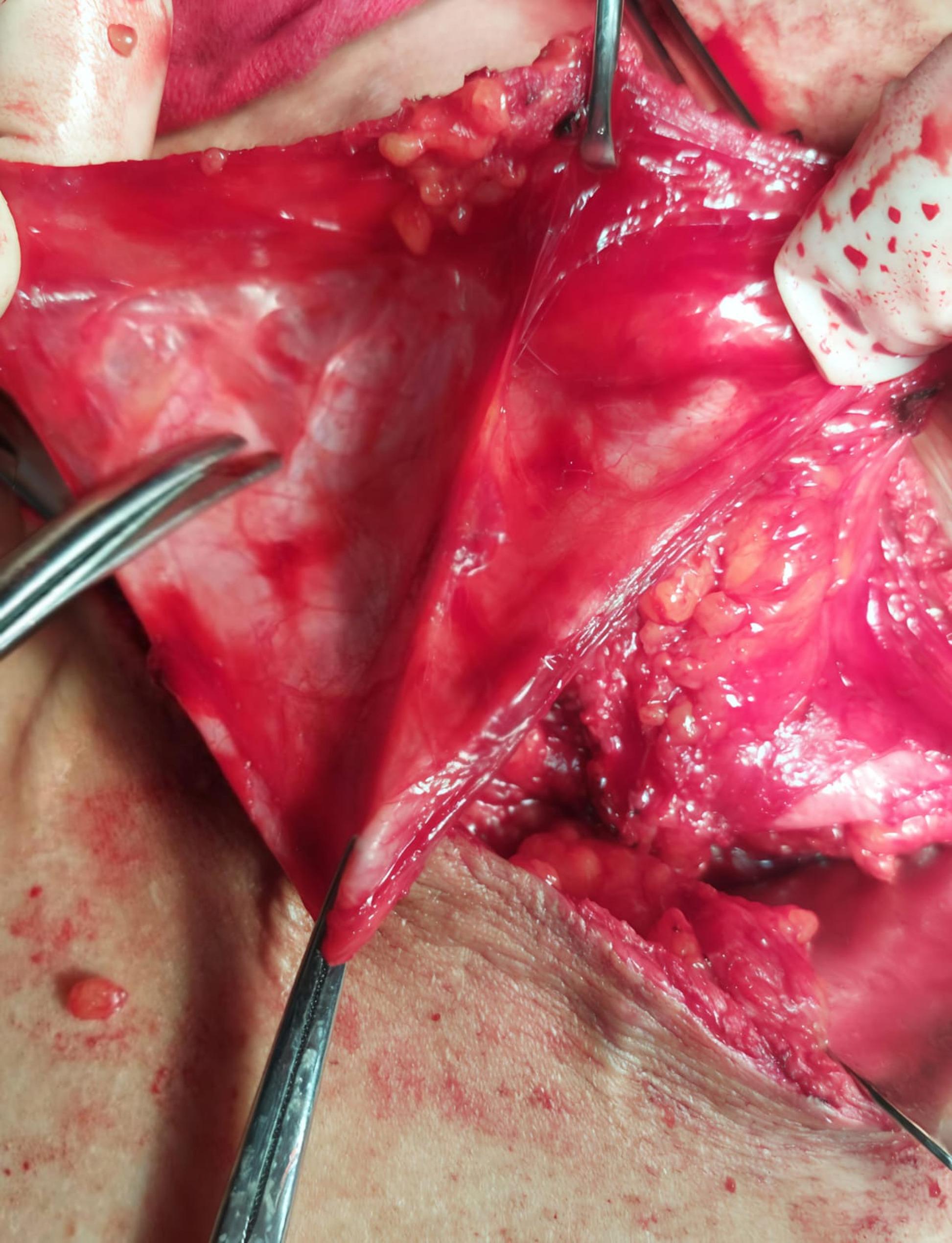
Dissection of the sac from the overlying muscles with creation of the preperitoneal pocket for the application of the mesh plug

The site of the hernia and the extent of hernial bulge were marked before the operation. Surgical procedures were performed under general anesthesia while the patient was in a lateral position (flank position) with elevating the bridge of the table to facilitate hyperextension of the surgical site in the lumbar region. An elliptical incision was performed to remove the thinned skin and scar. Upon accessing the muscular layer, the muscles were dissected and retracted to reveal the preperitoneal space where the peritoneum was dissected from the muscles without entering the peritoneum cavity (Fig. 2). with the upper limit of the dissection the costal margin and the lower limit is the iliac crest the anterior and posterior limits extend beyaud the atrophic muscles for at least two Cm. Any inadvertent breach in the peritoneal sac was stitched by Vicryle^®^ 3/0 sutures.

**Fig. 3:**
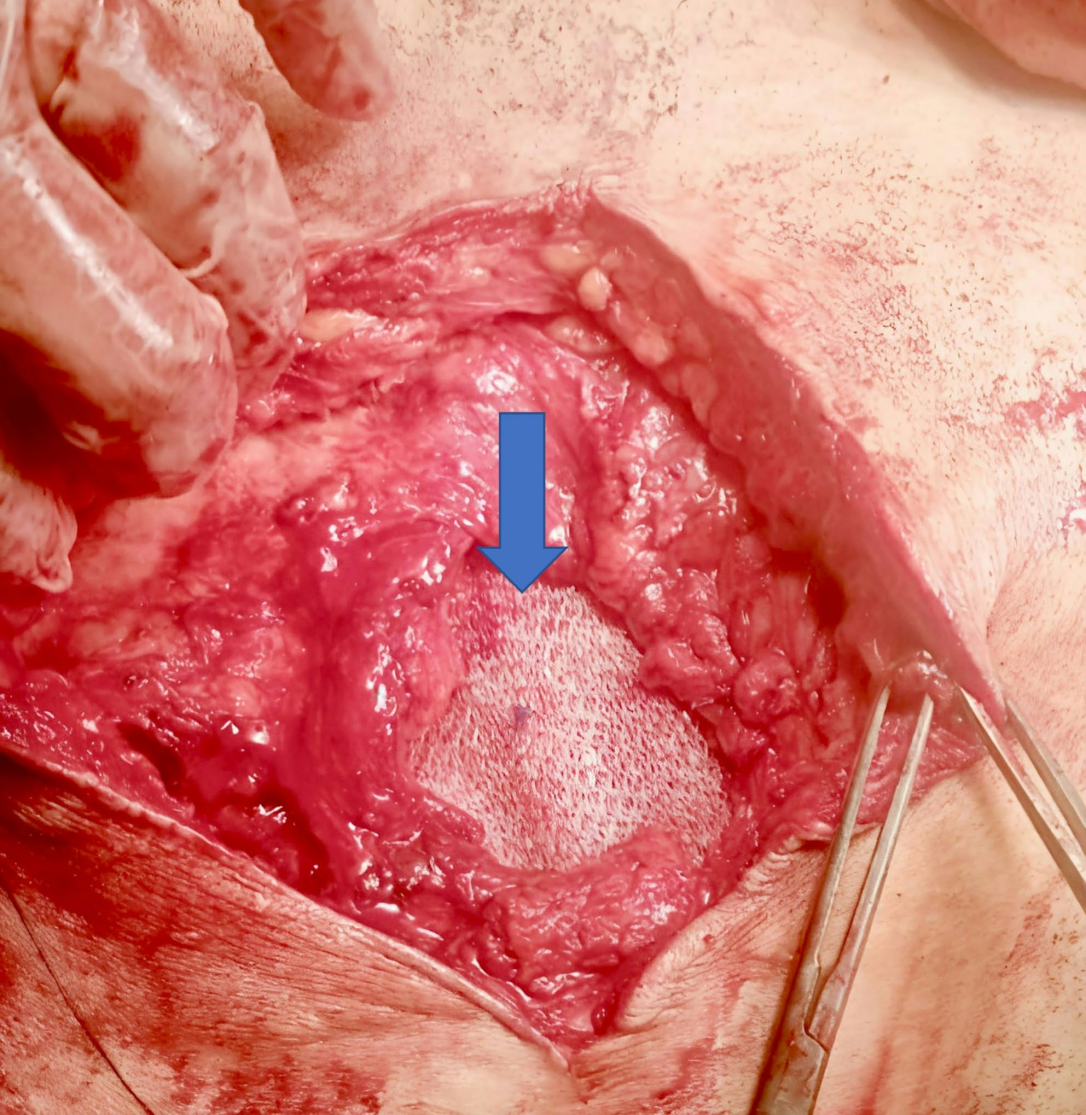
Three-layered polypropylene mesh plug placed extraperitoneally (arrow)

**Fig. 4:**
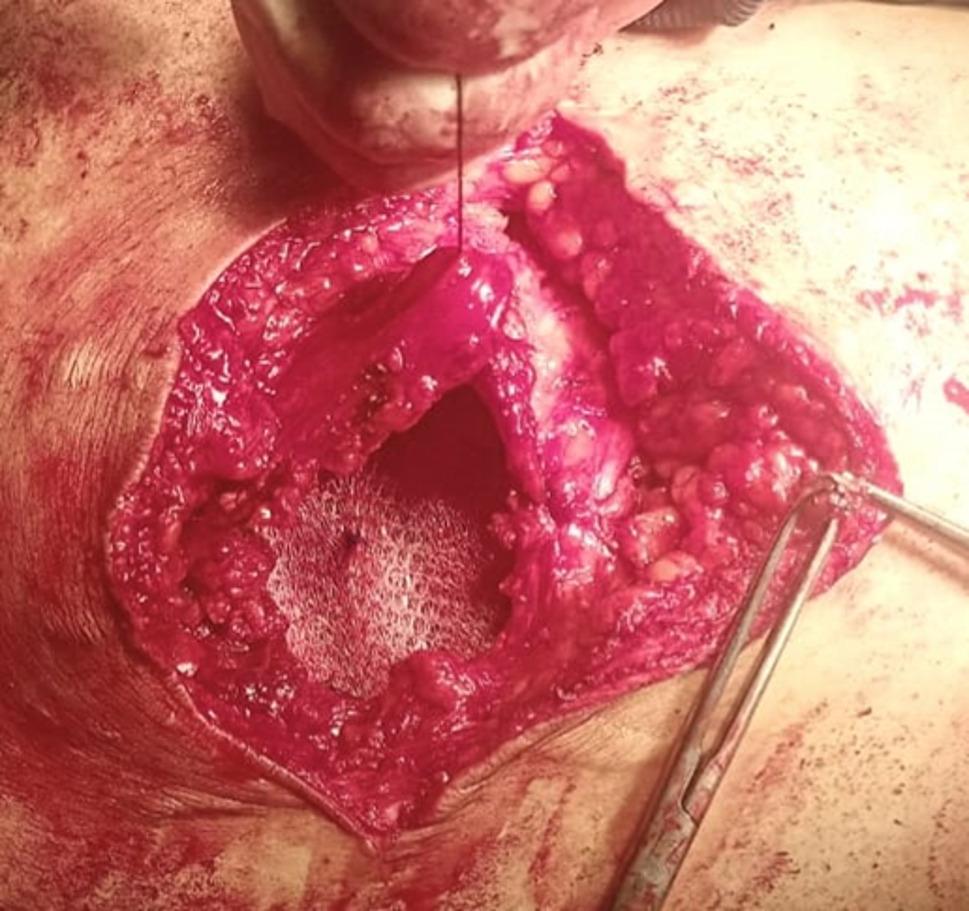
The muscle defect was closed using 0 Vicryl suture

**Fig. 5:**
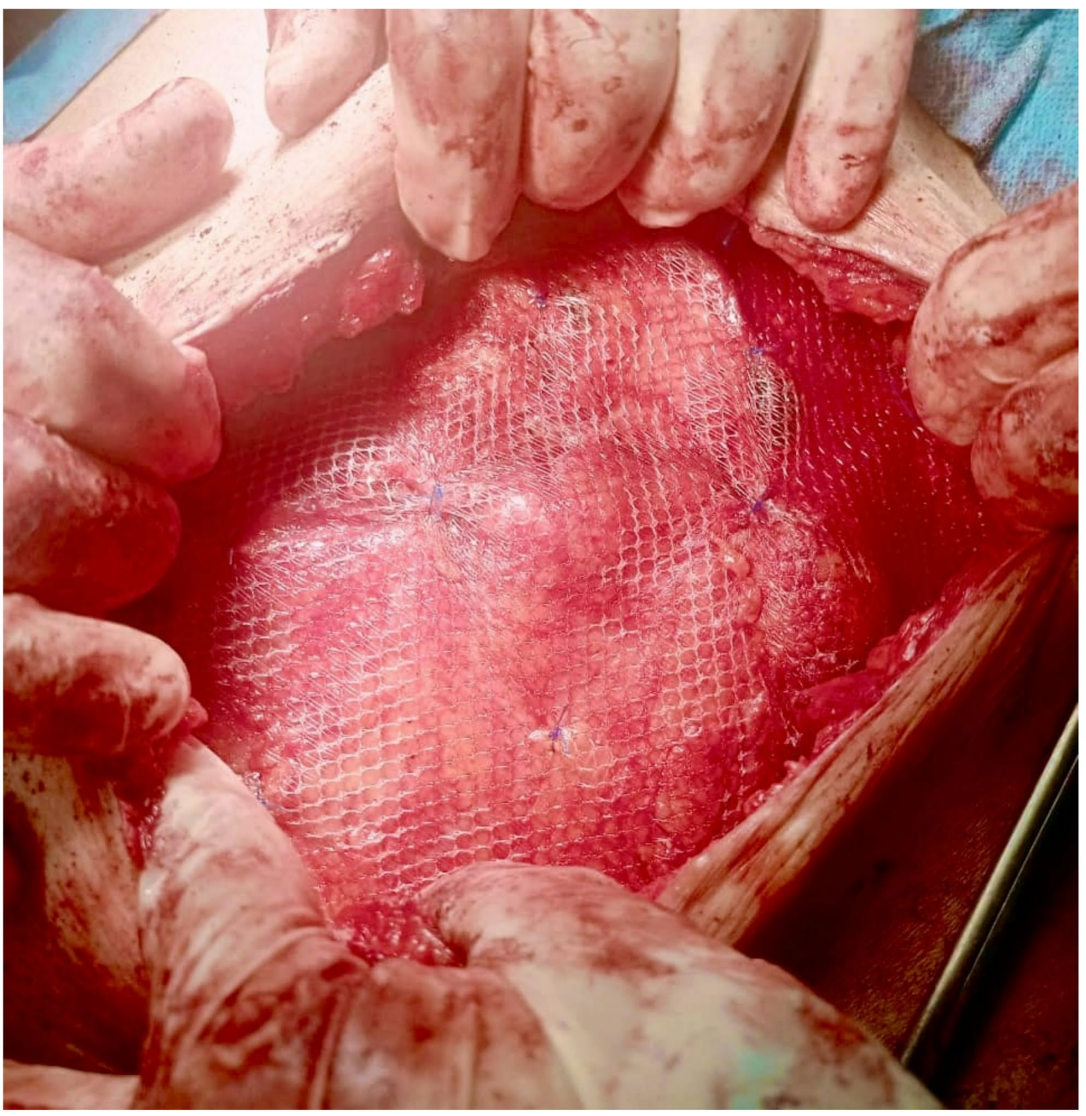
The onlay polypropylene mesh was thereafter positioned and affixed with at least 5 cm overlap beyond the muscle-sandwich technique

A 15 × 15 polypropylene light weight mesh sheet was folded twice to create a three-layer plug intended to occupy the preperitoneal space and reduce the sac. Two or three proline^®^ 2/0 stitches were used to secure the three-layered mesh plug together. The plug was anchored to the preperitoneal (sublay) plane with proline^®^ 2/0 to the adjacent muscle reflection (Fig. 3). The muscle was subsequently closed using 0 vicryl^®^ suture (Fig. 4). The onlay mesh was thereafter positioned and affixed with at least 5 cm overlap beyond the muscle repair (Fig. 5). Finally, the skin was closed over a suction drain.

### Follow-up

**Fig. 6:**
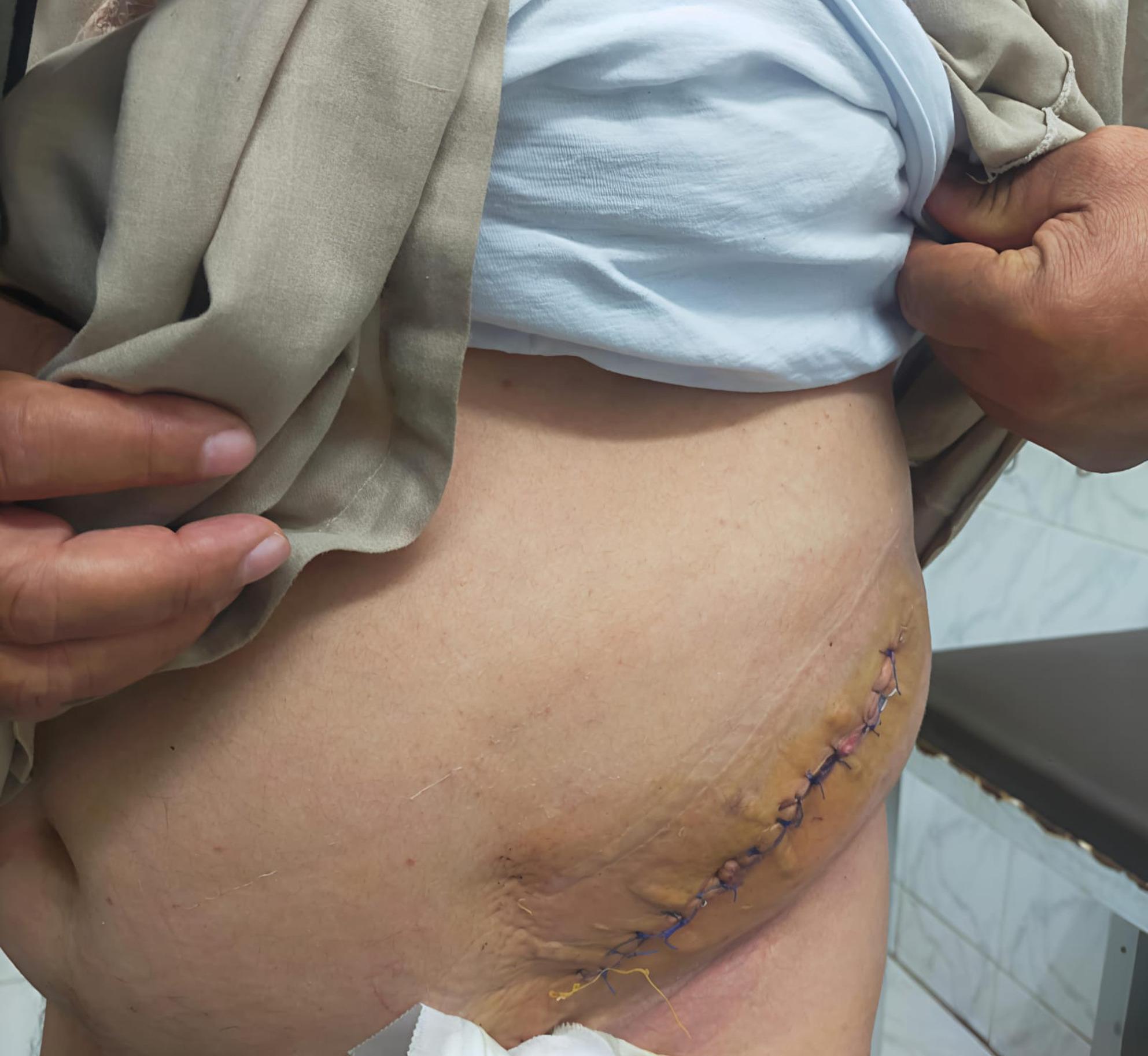
The patient is in a standing position during follow-up on post-operative surgical wound care in an outpatient clinic

The patients underwent follow-up for a minimum of six months postoperatively at the outpatient clinic weekly (Fig. 6). in the first month, then monthly for at least six months postoperatively. Any complications were recorded.

## Results

Ten patients were involved in this study, six males and four females, with a mean age of 50.5 ± 9.32. The main complaints were abdominal protrusion at a site of previous abdominal operation, both in 100% of cases. Pain was present in four patients (Tables 1 and 2).

**Table 1:** Gender, presentation and complications

			Frequency	Percent
Gender	Male		6	60
	Female		4	40
Symptoms	Abdominal bulge	Yes	10	100
	Abdominal pain	Yes	4	40
	Unsatisfactory sight	NO	5	50
		Yes	5	50
Indication of previous lumbar incision	Nephrectomy		3	30
	Stone ureter		7	70
Comorbidities	No comorbidity		5	50
	Smoking		2	20
	Hypertension		3	30
	Diabetes		3	30
	Ischemic heart disease		1	10
Complications	No complications		8	80
	Wound seroma		2	20
	Superficial wound ischemia		1	10

**Table 2:** Age, duration of hernia, hospital stay, operative time and defect size

	*N*	Minimum	Maximum	Mean	Std. Deviation
Age (years)	10	36	63	50.5	9.32
Duration of the incisional hernia (years)	10	1	8	4.3	2.11
Hospital stays (days)	10	2	7	4	1.7
Operative time (minutes)	10	65	94	76.4	11.86
Defect size (cm)	10	8	11	9.9	0.99

All the patients had previous surgery through a lumbar incision; the most frequent surgery was for ureteric stone (7 patients), while the other causes were for nephrectomy (3 patients) (Table 1).

50% of patients presented with comorbidities, including diabetes mellitus, smoking habits, ischemic heart disease, and hypertension. The defect size ranged from 8 to 11 in the longest diameter with the mean size of 9.9 ± 0.99 (Table 2).

The operative time was 76.4 ± 11.86 min while the hospital stay was 4 ± 1.7 days. Patients complicate seroma were 2/10 (20%) of patients, and one patient had a superficial wound ischemia.

No patients reported foreign body sensation or chronic pain during the follow-up period. No cases of hernia recurrence were detected during. All patients resumed normal daily activities within two weeks postoperatively and were advised to avoid heavy lifting for three months.

## Discussion

The majority of patients with lumbar hernia are presented as non-complicated cases, with only 9% categorized as surgical emergencies.

Lumbar incisional hernias are frequently diffuse, with difficulty determining the edges of the defect. According to reports, incisional lumbar hernia complicates about 12% to 23% of patients with a flank incision for retroperitoneal aortic aneurysms surgeries, and 7% of all retroperitoneal approaches. It presented with an uncomfortable and cosmetically displeasing flank “bulge” caused by laxity of the transversus and oblique abdominal wall musculature [10–12].

The surgical management of lumbar incisional hernia is challenging due to its relative rarity and the lack of standardized techniques for repair. Furthermore, lumbar incisions result in damage to the intercostal nerves, causing denervation-related muscle atrophy of the abdominal wall, which is clinically referred to as a “flank bulge” [13].

The only treatment option for these kinds of hernias is surgery. Muscle flaps from the gluteus maximus, and latissimus dorsi and facia lata were used to correct the majority of lumbar hernias in the past; however, the management had a very high rate of recurrence due to poor facial strength. Later, the surgeons used artificial polypropylene mesh [14].

Many patients report discomfort following the repair of incisional flank hernias,

and existence of a muscular protrusion at the site of the incision after surgery. So, many questions come up about the advantages of surgical intervention. Therefore, by using double mesh in our study, to reduce the likelihood of recurrence and improve the postoperative asymmetry.

This study evaluated the outcomes of the sandwich mesh hernioplasty technique for lumbar incisional hernia repair in ten patients. To reduce the recurrence rate, our technique combines the method described for the pair of lumbar incisional hernias, the onlay mesh hernioplasty, with an additional mesh patch that was deployed into the preperitoneal space. This dual-layer reinforcement evenly distributes intra-abdominal pressure, hence reducing the risk of recurrence.

Hospital stay of four days is standard for open abdominal wall repairs. The complication rate was acceptable; two patients (20%) developed seroma, and one (10%) had superficial wound ischemia which was managed conservatively. Seroma formation is the most frequently reported complication after open mesh hernia repair, with rates varying from 5% to 30% [15]. One patient developed a wound infection characterized by fever and erythema, which responded to therapy with topical antibiotics, gentamicin cream, and systemic antibiotics.

Although the early outcomes are promising, the present study is limited by its small sample size and relatively short follow-up duration. Sandwich mesh hernioplasty for lumbar incisional hernias needs larger cohorts and longer follow-up to prove its long-term efficacy.

## Conclusion

The sandwich mesh repair technique represents a potentially feasible surgical option for lumbar incisional hernia repair, demonstrating satisfactory early outcomes and acceptable complication rates.

## Limitations

Although the sandwich mesh technique demonstrated satisfactory early outcomes and acceptable complication rates, the small sample size and limited follow-up represent significant limitations. Further prospective multicenter comparative studies with large sample sizes and longer follow-up are required to confirm the long-term efficacy and durability of this approach.

## Data Availability

The datasets generated during the current study are available from the corresponding author on reasonable request.

## Abbreviations

NCT: National Clinical Trial
CT: Computed Tomography
HbA1c: Hemoglobin A1c (glycated hemoglobin)
IRB: Institutional Review Board
US: Ultrasound
